# Liquid phase exfoliation of graphene using ammonia as an easy-to-remove additive in low-boiling organic-water co-solvent suspensions

**DOI:** 10.1038/s42004-025-01517-y

**Published:** 2025-05-23

**Authors:** Martin Nastran, Paul Peschek, Izabela Walendzik, Jakob Rath, Bernhard Fickl, Jasmin S. Schubert, Wolfgang Ipsmiller, Andreas Bartl, Gerd Mauschitz, Gabriel Szabo, Richard A. Wilhelm, Jochen Schmidt, Dominik Eder, Bernhard C. Bayer

**Affiliations:** 1 Technische Universität Wien (TU Wien), Institute of Materials Chemistry, Getreidemarkt 9/165, Vienna, Austria; 2 Wrocław University of Science and Technology, Department of Process Engineering and Technology of Polymer and Carbon Materials, Gdańska 7/9, Wrocław, Poland; 3 Technische Universität Wien (TU Wien), Institute of Chemical, Environmental and Bioscience Engineering, Getreidemarkt 9/166-1, Vienna, Austria; 4 Technische Universität Wien (TU Wien), Institute of Applied Physics, Wiedner Hauptstrasse 8-10/134, Vienna, Austria; 5 carbon-solutions Hintsteiner GmbH, Kirchengasse 1, Mürzhofen, Austria

**Keywords:** Synthesis of graphene, Two-dimensional materials

## Abstract

Graphene nanosheets from suspensions are key to applications such as in printable films, battery/supercapacitor electrodes, fillers in composite materials or catalyst supports. We present a straightforward method for achieving high-concentration and long-term stable graphene suspensions by liquid phase exfoliation (LPE) via a combination of ammonia (NH_3_) as an easily removable additive together with low-boiling point, benign organic-water co-solvent mixtures as suspension media. We find that the addition of small amounts of NH_3_ as an additive drastically improves the obtainable LPE graphene concentrations by up to 2 orders of magnitude for a wide range of organic-water co-solvent mixtures including with isopropanol, methanol, ethanol, 1-propanol, tetrahydrofuran, acetonitrile, acetone, ethylene glycol and tert-butanol. With our approach we readily reach current benchmark graphene concentration values of ~180 mg·L^-1^ that are normally only obtainable using hard-to-remove high-boiling-point and hazardous standard solvents like dimethylformamide and 1-methyl-2-pyrrolydone or with hard-to-remove surfactants. Notably, NH_3_ as an additive is highly volatile and thus, as we show, easily removable without degrading the produced high quality graphene nanosheets.

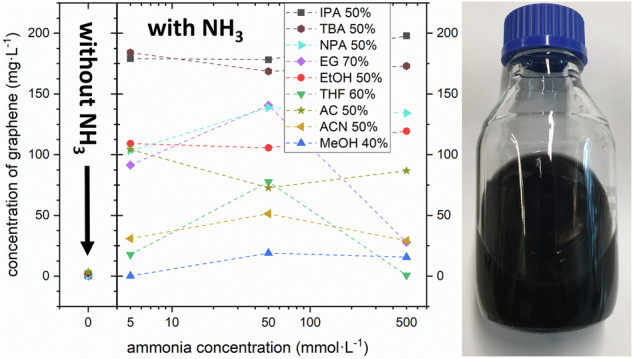

## Abstract (web summary)

Liquid phase exfoliation (LPE) is a common technology used for graphene synthesis, however, the optimization of liquid suspension medium remains challenging. Here, the authors report a combination of ammonia (NH_3_) as an easily removable additive together with low boiling point organic-water co-solvent mixtures as suspension media to achieve high-concentration and long-term stable graphene suspensions by LPE, reaching current benchmark graphene concentration values of ~180 mg·L^-1^.

## Introduction

Graphene possesses a unique combination of exciting properties that make it promising for a wide variety of applications^1,2^. Form factors of interest for graphene for its various applications range from monolayered films with cm+ lateral extents for optoelectronics and barrier layers (as typically produced bottom-up in chemical vapor deposition^3,4^) to two-dimensional (2D) graphene “powders”. The latter is comprised of few-layer graphene 2D nanosheets that typically have thickness distributions from 1 to ~20 layers and lateral sizes in the range of hundreds of nm up to several µm^5,6^. These graphene “powders” have a plethora of potential applications as 2D building blocks for, e.g., printable films^7^, electrode materials in batteries or supercapacitors^8^, fillers in composite materials or heterogeneous catalyst components or supports^1,2^. These graphene “powders” are typically produced in a top-down fashion by exfoliation from bulk graphite^5^. For this top-down synthesis, liquid phase exfoliation (LPE) has established itself as a frontrunner synthesis technology^9^. LPE relies on bringing the bulk starting material in an appropriate liquid suspension medium (often termed “solvent”) and coupling energy into the system to facilitate exfoliation of the bulk material into the few-layered 2D nanosheets^5,10^. The required energy can be coupled via ultrasonication, tip-sonication, or shear forces from, e.g., shear mixers. Key to LPE is the appropriate choice of suspension medium (solvent), whereby critically, the solvent must facilitate not only exfoliation into 2D nanosheets but also ensure a stable dispersion of the 2D nanosheets, i.e., inhibit restacking of produced 2D sheets for prolonged times even after the energy input ceases^5,10^. Additionally, an ideal solvent must be readily removable from the 2D nanosheets to bring them into dry 2D powder form or readily exchangeable to incorporate the 2D nanosheets into other chemical environments, all ideally without significant restacking in the process. To date, no such ideal solvent has yet been identified^11,12^, and the search for appropriate solvents for graphene LPE (and LPE of many other 2D materials) is still ongoing.

In general, for LPE, the solvents can be divided into organic and aqueous solvent systems: for organic solvents, LPE of graphene can often be performed in the neat organic solvent, although commonly used solvents like dimethylformamide (DMF, boiling point (b.p.) 153 °C) and 1-methyl-2-pyrrolydone (NMP, b.p. 203 °C) often have a high-boiling point (hindering drying^13^ and solvent exchange) and are hazardous to human health^14^. High-quality graphene can also be obtained by using mixtures of organic solvents, but the concentrations here are usually lower or require longer sonication times^15^. Lower boiling point organic solvents with a more benign health hazard profile would, therefore, be highly desirable for the LPE of graphene and 2D materials.

In contrast to neat organic solvents, aqueous LPE of graphene does not work in neat water without the addition of suitable surfactants, which are required to allow dispersion of the 2D nanosheets. These surfactants are alkaline salts or polymers, such as sodium dodecylbenzene sulfonate (SDBS), sodium cholate, or poly(vinyl pyrrolidone) (PVP)^16–19^. These surfactants are adsorbed onto the 2D nanosheet surface by noncovalent interactions and prevent the nanosheets from reaggregation by electrostatic and/or steric repulsion^20^. However, due to their high adsorption affinity, these surfactants can lead to unwanted modifications of the graphene surface and are very challenging to remove post-LPE^17^, which can be particularly hindering in, e.g., catalysis and electrical applications. In particular, in many LPE systems, the concentration of the surfactant can be close to the final 2D nanosheet concentration in the product suspension^16,21^, which significantly reduces the achievable purity of the 2D nanosheets^19^. Hence, there is a need to develop easily removable LPE-stabilization additives or surfactants for graphene and 2D materials LPE.

Herein, we bridge these challenges in graphene LPE by using a combination of an easy-to-remove additive, namely ammonia (NH_3_), together with relatively low-boiling point, benign organic-water co-solvent mixtures. We demonstrate that the addition of NH_3_ as an additive drastically improves the obtainable LPE graphene concentrations by up to two orders of magnitude for a wide range of organic-water co-solvent mixtures. Notably, NH_3_ as an additive is highly volatile and thus, as we show, easily removable without degrading the produced high-quality graphene 2D nanosheets.

Comparing with previous literature, we note that Arao et al. have previously tangentially mentioned a beneficial effect of NaOH and NH_3_ addition to graphene LPE^22^ and later reported that ammonium carbonate ((NH_4_)_2_CO_3_) can be used as an additive for organic solvents to improve graphene LPE and reduce agglomeration^23^. NH_3_ has also previously been shown to enhance LPE in neat water^24,25^. Building on these highly useful prior findings, here we, for the first time, deliberately and directly introduce NH_3_ as the sole additive during graphene LPE in a large range of organic solvent/water mixtures. We show that this NH_3_ addition has a highly beneficial effect on graphene LPE for a very wide selection of organic components in organic-water co-solvent mixtures, including with isopropanol (IPA), methanol (MeOH), ethanol (EtOH), 1-propanol (NPA), tetrahydrofuran (THF), acetonitrile (ACN), acetone (AC), ethylene glycol (EG) and tert-butanol (TBA), with ammonia being soluble in many organic solvents. This drastically underlines the generality of this approach. Combined, our work demonstrates a facile route to improved graphene LPE yield, thus providing a framework for simpler and safer 2D graphene LPE development.

## Results and discussion

We first describe our considerations in selecting the LPE solvent systems to be used. Key considerations for us were low-boiling points to allow easy solvent removal/exchange and a benign health hazard profile. A key factor complicating this search is that the best solvent for LPE of a given 2D material has been found to be highly dependent on the nature of the 2D material^11^. No generally applicable predictive theory for finding ideal solvents or solvent mixtures for given 2D materials has yet been identified. Instead, the exploration of new solvents is largely based on empirical trial and error. However, as a somewhat general guideline among many influencing factors, it has been found that for LPE solvents, it is beneficial if the surface energy of the solvent matches that of the 2D material in order to obtain good 2D LPE yields^5,14^. For the particular case of graphene, it has been shown that for neat solvents, the most effective ones for the exfoliation of graphite have a surface tension close to 40 mN m^-1^ (refs. ^14,26^). Since the surface energy of water (72.8 mN·m^-1^) is too high to exfoliate graphite directly efficiently when following this logic, a mixture with common organic solvents, which possess a lower surface energy, has been reported to lead to an optimal mixture when mixed in different ratios^27–29^. We, therefore, test here this co-solvent strategy comparing binary mixtures over a wide composition range of a large number of common organic solvents with water, as well as the neat organic solvents and neat water as reference. The tested co-solvents and their corresponding surface tension values are summarized in Table 1. With these identified co-solvent systems, the effect of NH_3_ addition as an additive to these co-solvent and neat systems is then studied by adding a series of small NH_3_ concentrations (0 mmol·L^-1^ reference baseline and 5, 50, and 500 mmol·L^-1^ series) to the various co-solvent systems.

**Table 1:** Overview of investigated solvents^14,47–49^

Abbreviation	Name	Linear formula	Surface energy (mN·m^-1^)	Boiling point (°C)
TBA	tert-Butanol	(CH_3_)_3_COH	20.7	83
EtOH	Ethanol	CH_3_CH_2_OH	22.1	78
MeOH	Methanol	CH_3_OH	22.7	65
IPA	Isopropanol	(CH_3_)_2_CHOH	23.0	83
NPA	1-Propanol	CH_3_CH_2_CH_2_OH	23.7	97
AC	Acetone	CH_3_COCH_3_	25.2	56
THF	Tetrahydrofuran	(CH_2_)_4_O	26.4	66
ACN	Acetonitrile	CH_3_CN	29.0	82
EG	Ethylene glycol	HOCH_2_CH_2_OH	47.7	197
	Water	H_2_O	72.8	100
DMF	N,N-Dimethylformamide	C_3_H_7_NO	37.1	153
NMP	1-Methylpyrrolidin-2-one	C_5_H_9_NO	40.1	203

Note that none of the studied, low-boiling mixtures exhibits an increase of the boiling point compared to water (boiling point: 100 °C), as no mixture exhibits a negative azeotrope^50,51^. Also, all studied mixtures have boiling points lower than the typical benchmark solvents DMF (153 °C) and NMP (203 °C). See also Supplementary Table 1.

**Fig. 1:**
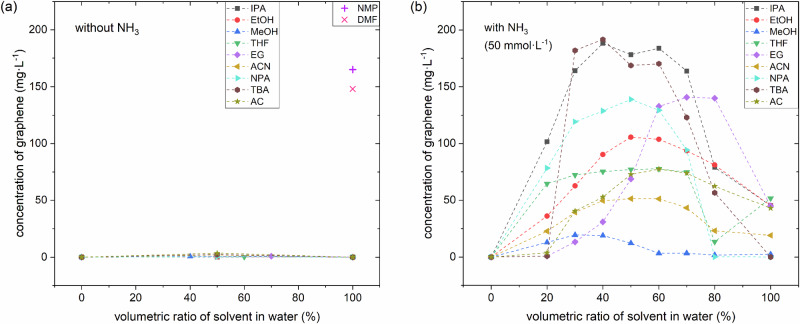
Exfoliation of graphene in different (co-)solvents without and with NH_3_. Concentration of graphene as measured by UV–Vis after LPE incl. centrifugation for various organic-water mixtures at concentrations of organic solvent from 0 to 100% without NH_3_ addition (**a**) and with 50 mmol·L^-1^ NH_3_ addition during LPE (**b**).

We then test all the various co-solvent mixtures for graphene LPE under otherwise constant exfoliation (6 h in bath ultrasonicator) and centrifugation conditions (30 min at 5500 rpm) using a simple commercial ultrasonic bath and a standard mild centrifugation protocol. The obtained graphene concentrations after centrifugation are then measured using UV–Vis spectroscopy^14^. In addition, for selected samples, the graphene nanoflakes obtained are further characterized using atomic force microscopy (AFM), transmission electron microscopy (TEM), and X-ray photoelectron spectroscopy (XPS). Further details of the methods are given in the “Methods” section below and in the Supplementary Information. Figure 1 plots our key findings. We first find in Fig. 1a that for our exfoliation protocol *without* NH_3_ addition, the obtained graphene concentrations remain low at well below <10 mg·L^-1^ for neat water and all neat organic solvents as well as selected binary organic solvent-water mixture concentrations with ~50% solvent content. This is significantly lower by two orders of magnitude compared to benchmark, high-boiling point solvents like DMF and NMP, which we also investigated as benchmarks in Fig. 1a. This implies that the here selected co-solvent mixtures alone do not significantly improve graphene LPE concentration under our conditions.

In Fig. 1b we show the effect of adding a small amount of NH_3_ (50 mmol·L^-1^) to the various neat solvents and the binary organic solvent-water mixtures (measured at more closely spaced concentration intervals than in Fig. 1a). For the neat water and the neat organic solvents, the effect of NH_3_ addition is rather small in terms of increased graphene concentration (negligible increase for neat water, maximum increase by factor of 10 for some neat organic solvents).

In very drastic contrast, however, for the binary organic solvent-water mixtures, we obtain increases in graphene concentration of up to two orders of magnitude via the addition of NH_3_. Figure 1b shows that for most mixtures, this increase is most pronounced around ~50% organic content, yielding up to 180 mg·L^-1^ graphene concentration. These values are well comparable with the concentrations resulting from the use of DMF or NMP (Fig. 1a) or concentrations obtainable using water with hard-to-remove surfactants like SDBS, cholates, or PVP^16,17^. We emphasize that we clearly see this beneficial effect for a very wide range of organic co-solvents, which underscores the generality of our approach of using NH_3_ to improve LPE graphene concentration.

We also note that for several of the here investigated co-solvent mixtures with NH_3_, the dependence of the highest graphene concentration on the solvent mixture forms a broad plateau (i.e., not a narrow peak). This suggests that the highly advantageous effect of NH_3_ addition to the organic-water co-solvents is a very robust phenomenon over a reasonably wide co-solvent composition range, which eases technical implementation. Furthermore, the highest concentrations are obtained for mixtures of IPA-water for 40% to 60%, which is also technologically hugely advantageous since IPA is very easy to handle, safe, and a cheap organic solvent.

For most organic-water mixtures (IPA, EtOH, NPA, THF, ACN, AC, TBA), the highest concentration values with NH_3_ addition are obtained around ~50% organic solvent to water ratio. We suggest that this is related to the observation from Table 1 that all of these organic solvents have surface energies ~20 to 25 mN·m^-1^. From a simple rule of mixtures, their ~50% mixtures with water (surface energy: 72.8 mN·m^-1^) are expected to have a surface energy of ~45 mN·m^-1^, which is close to the surface energy of graphite ( ~ 40 mN·m^-1^). Such a match of surface energies has previously been identified as being beneficial to LPE yield^16,26,30^. Our findings here, thus, stress that such surface energy match reasoning can also be a guideline for co-solvent mixture selection when using the reported NH_3_ additive. We underscore, however, that all organic-water solvent mixtures without NH_3_ showed only much lower LPE yield alone, irrespective of surface energy (Fig. 1a). This again underscores the here identified key role of NH_3_ addition to increasing graphene LPE concentration in our study.

We also note that two solvents show distinct differences in this behavior of best graphene yield at ~50% organic-water mixture: EG and MeOH. For EG, the highest graphene yield is found at ~70%. This can again be ascribed to surface energy matching, since EG has a distinctly higher surface energy (47.7 mN·m^-1^), thus requiring less water to match graphite’s surface energy. For MeOH the best relative graphene concentration was obtained at ~30%. Since MeOH has a surface energy of 22.7 mN·m^-1^, which is in line with the majority of the above-discussed solvents, from pure surface energy consideration, the best concentration would also be expected at ~50%. This suggests that further factors other than surface energy are also of importance in solvent selection for NH_3_-assisted LPE, which at present remains unknown for MeOH with NH_3_. This is in line with the state of the general incomplete understanding of general LPE solvent chemistry on other systems without NH_3_ addition^11^.

**Fig. 2:**
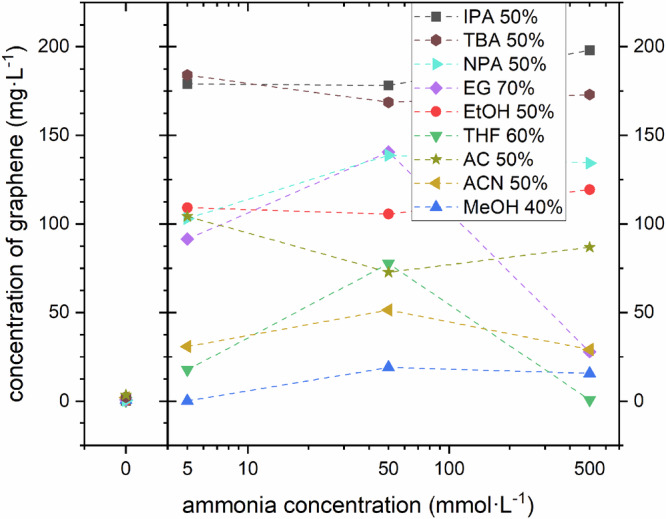
Exfoliation of graphene in different co-solvents for different NH_3_ concentrations. Concentration of graphene as measured by UV–Vis after LPE incl. centrifugation for various organic-water mixtures at concentrations optimized with respect to the highest graphene yield from Fig. 1b as a function of NH_3_ additive concentration 0 (baseline), 5, 50, and 500 mmol·L^-1^ NH_3_.

In Fig. 2 we further explore how compositionally wide the beneficial effect of NH_3_ addition to our organic-water co-solvents is. To this end, we chose the highest graphene concentration yielding organic-water mixture concentrations for each organic solvent from Fig. 1b, while varying the concentration of NH_3_ logarithmically from 0 (baseline), 5, 50 to 500 mmol·L^-1^ NH_3_. We find that despite the logarithmic variation of NH_3_ concentration, the graphene LPE yield only slightly varies as a function of NH_3_ concentration for the optimized organic-water mixtures. This clearly shows that the addition of NH_3_ has a compositionally very wide effect, useable over a wide NH_3_ concentration range. This is of significant technological benefit, as close control over NH_3_ additive/surfactant concentration is not necessary to benefit from the NH_3_ stabilization in the various co-solvents.

To show the visual appearance of the graphene during the various stages of the LPE process, i.e., directly after sedimentation, after different sedimentation times, and after centrifugation, photographs at the various stages are shown in Supplementary Fig. 1 for the optimized 50% IPA-water with 50 mmol·L^-1^ NH_3_ alongside its neat components and the neat co-solvent mixture without NH_3_. This data again visualizes that without NH_3_ addition, non-centrifuged suspensions sediment, and after centrifugation only for the co-solvent with NH_3,_ significant graphene remains in the suspension (in line with the quantitative concentration measurements in Figs. 1 and  2).

**Fig. 3:**
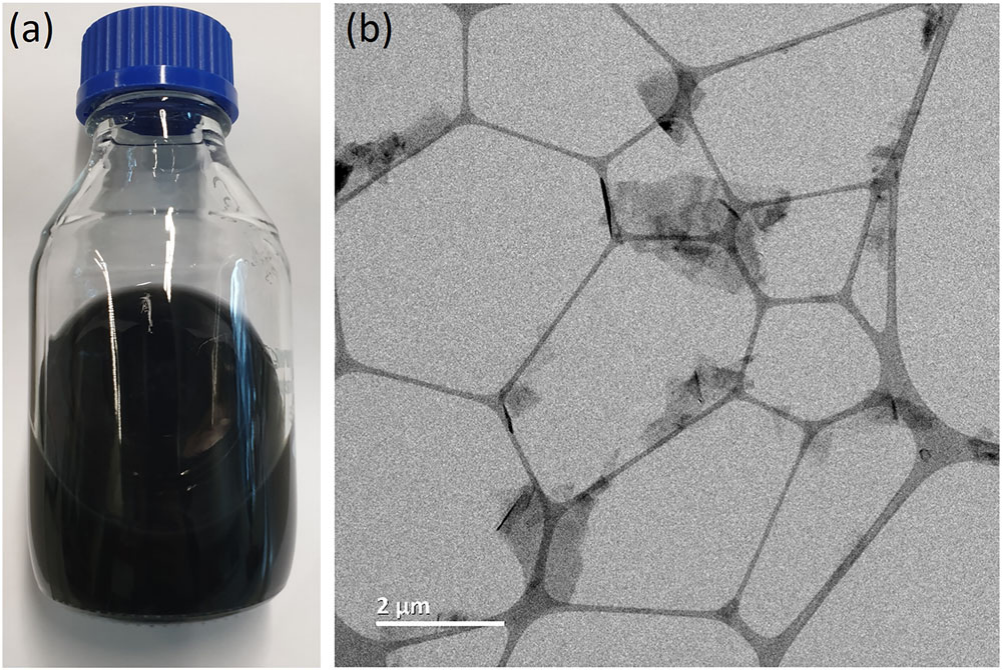
Macroscopic and nanoscopic characterization of produced graphene. **a** Photo of IPA-water 50% with 50 mmol·L^-1^ NH_3_ suspension in vial after 6 months storage. **b** Bright-field TEM overview image of IPA-water 50% with 50 mmol·L^-1^ NH_3_ suspension after drying, showing several graphene flakes on lacey carbon support. Note the observed high electron transparency of the flakes is indicative of low thickness.

We also note that the obtained optimized organic-water with NH_3_ additive suspensions remain stable over months without any appreciable signs of loss of concentration, agglomeration or sedimentation (see example image in Fig. 3a). This observed long-term stability of our graphene suspensions is of high technical benefit.

For the IPA-water 50% with 50 mmol·L^-1^ NH_3_ samples, which are amongst the highest obtained graphene concentrations in this study, we investigate the obtained flakes microscopically in additional detail. TEM images in Fig. 3b and Supplementary Fig. 2 and AFM images in Supplementary Fig. 3 reveal that we obtain few-layer graphene nanoflakes with thicknesses in the range of 1 nm to 10 nm, corresponding to ~3 to ~30 layers thickness. Selected area electron diffraction (SAED) in Supplementary Fig. 2a confirms that our obtained few-layer graphene nanosheets are of high crystalline quality, as evidenced by very sharp six-fold symmetric spots in the SAED patterns^14^. Lateral flake sizes are found with an average of ~1 µm with a maximum of up to ~3 µm (see lateral flake size histogram in Supplementary Fig. 2c). Both thickness distribution and lateral sizes are comparable to similar work with benchmark NMP and established water/surfactant systems^31^.

We now briefly discuss possible mechanisms behind our results: With respect to our use of water-organic co-solvent mixtures, prior work suggested that the use of water and a co-solvent modifies the surface tension to a for LPE reasonable good value around 40 mN·m^−1^, which improves wettability of graphite particles and makes the exfoliation possible^12,14^. For instance, Li et al. showed that a equivolumetric solution of IPA-water ruptured pristine graphite flakes into flatter pieces in contrast to a water control^32^. This is particularly interesting because poor dispersion of graphene does not necessarily mean poor yield of few-layer graphene during exfoliation^33^.

With respect to possible mechanistic reasons behind the observed highly beneficial effect of NH_3_ addition on graphene concentration, we note that we here do not aim to conclusively identify the underlying mechanisms in this report but rather place our new experimental findings in the context of the current understanding of alkaline additives in graphene LPE^34^. As the first argument, the stabilization mechanism for the graphene solutions is suggested to be based on electrostatic repulsion. The surface of few-layer graphene is negatively charged in many solvents (presenting a Zeta potential of ‒30 to ‒40 mV)^35^, whereby tuning the Zeta potential via modification of the pH can reduce restacking and agglomeration^24^. In order to obtain more detailed information about the influence of NH_3_ on this mechanism, we performed Zeta potential measurements of different dilutions of the best working graphene suspensions exfoliated in 50% IPA-water with 50 mmol·L^-1^ NH_3_. The measurements of these highly stable graphene suspensions yielded Zeta potentials between ‒27.1 and ‒28.1 mV, which are values close to the above-reported range, which is encouraging since Zeta potentials of +/‒ 30 mV are often considered sufficient for stabilizing colloidal dispersions among particle technology in general. Consequently, these values indicate a beneficial influence of electrostatic stabilization of the graphene flakes. This suggests that the here employed ammonia hereby optimizes the Zeta potential and, hence the stability of the exfoliated graphene^24^. Additionally, via the NH_3_ a “wedge” effect on freshly formed fracture surfaces can also be suggested during exfoliation^24,36,37^.

The importance of the presence of NH_3_ on the dispersion stability of the graphene suspensions is also further experimentally confirmed in a further test experiment in Supplementary Fig. 4: While with NH_3_ addition, our graphene suspensions remain stable over months (see above Fig. 3a), with acidic neutralization of the ammonia the graphene suspension becomes immediately unstable (Supplementary Fig. 4). The result of NH_3_ neutralization is a visible agglomeration of the graphene flakes to macroscopic aggregates within a few seconds, which gravitationally sediment over the course of a few hours, leaving only a clear supernatant once NH_3_ is neutralized. This again emphasizes the key role of NH_3_ in making our graphene suspension stable in the organic-water mixtures.

A key presumption for choosing NH_3_ as an additive for graphene LPE was that the NH_3_ is highly volatile and hence is expected to be readily removable from the graphene nanosheets by, e.g., a mild evaporation step (unlike classical surfactants like SDBS, sodium cholate or PVP)^16–18,21,38^. To confirm this hypothesis, we measured XPS on dried (60 °C in air) graphene nanoflakes from IPA-water 50% with 50 mmol·L^-1^ NH_3_ (Supplementary Fig. 5). The detail C1s spectrum (Supplementary Fig. 5b) exhibits a main peak at 284 eV assigned to sp^2^ carbon atoms^39–42^. The C1s spectrum further shows the typical asymmetric spectral shape with the π-π* shake-up component ( ~ 292 eV) of graphitic carbon^41,43^. The remaining minor C1s components are assigned to sp^3^-like flake edges and adventitious carbon adsorption, typical for graphene produced via the LPE route^44–46^. Hence the C1s spectrum is consistent with high-quality graphene few-layer flakes and in line with the high structural graphene quality seen in SAED (Supplementary Fig. 2a). With respect to NH_3_ presence in the dried film, the N1s spectra (Supplementary Fig. 5d) reveals only a negligible residual amount of N signal in the final dried product, close to the detection limit of the XPS ( < < 1 atom-%). This thereby fully confirms our hypothesis that NH_3_ is easily removed via simple ambient drying from the LPE graphene, after enabling high-concentration and long-term stable LPE of high-quality graphene nanosheets.

## Conclusions

In conclusion, we demonstrated LPE of suspensions of graphene nanosheets at high graphene concentrations and long-term stability (>6 months) in different organic-water co-solvent mixtures with NH_3_ as an easy-to-remove additive. Importantly, most of the screened organic solvent mixtures have a relatively low boiling point of ≤100 °C and a benign health hazard profile. For most organic solvents, optimal exfoliation was obtained using a mixture of organic solvent with around 50% water, with an addition of small amounts of NH_3_ being critically necessary to improve exfoliated graphene concentration up to two orders of magnitude. We thereby demonstrate a high-yield graphene LPE synthesis via a very readily removable additive (NH_3_) in a wide range of solvent mixtures incl., low-boiling point and benign mixtures, that readily reaches current benchmark graphene concentration values of ~180 mg·L^-1^ (that are normally only obtainable for hard-to-remove high-boiling and hazardous standard solvents like DMF or NMP or with hard-to-remove surfactants). Thereby, our findings contribute to the ongoing search for simpler, easily removable, and safer solvent systems in graphene and 2D materials LPE development.

## Methods

As precursor material, pristine graphite flakes (325 mesh, <45 µm) were purchased from Alfa Aesar. Aqueous ammonia solution (17%), NPA, IPA, AC, MeOH, EtOH, THF, EG, TBA, ACN, NMP, and DMF were analytical quality and used without additional purification. Only deionized water was used from an in-house deionization system. To ensure that possible trace contaminations in the water did not influence LPE results, for selected sample combinations also reference measurements with ultrapure benchmark MilliQ water were undertaken. These yielded the same results with the MilliQ as with the otherwise used in-house water, ensuring that possible trace contaminations in the water are no significant factor in the results of this study. We have furthermore also tested the generality of our approach as a function of various graphite sources (as in these different impurities could potentially also influence results). As shown in Supplementary Fig. 6, we test beyond our otherwise used pristine graphite flakes (325 mesh, <45 µm) from Alfa Aesar four additional graphite sources (natural UF4 from Graphit Kropfmühl GmbH, TIMCAL TIMREX® KS6 Synthetic Graphite, TIMCAL TIMREX® KS75 Primary Synthetic Graphite, and TIMREX® SFG 75 Graphite). Fully underscoring the generality of our results, we find that all these additional sources yield similarly successful and stable exfoliation with our optimized 50% IPA-water with 50 mmol·L^-1^ NH_3_ recipe. This further underscores the generality of our exfoliation strategy beyond any particular graphite precursor source.

For the LPE process, first pristine graphite flakes (300 mg) were added into screw cap exfoliation vials (20 mL total capacity). The solvent mixtures (combined volume 15 mL) were added first, followed by 5, 50, or 500 mmol, respectively, of the aqueous ammonia solution. The vial caps were sealed using tape to prevent loosening and unscrewing of the caps during sonification. Exfoliation was performed by using a low-power sonification bath (Bandelin Sonorex Digitec DT 156 BH (9,0)) filled with water. The sample vials were sonicated for 6 h each. To ensure reproducibility, the ultrasonic bath was constantly kept at 2–7 °C via the addition of small portions of ice to the water during all experiments. Additionally, every sample position was cycled between bath positions uniformly during each run to ensure equal ultrasonication regardless of hot spots in the bath. As a reference check between runs of various compositions, an additional reference sample (50% IPA in H_2_O + 50 mmol·L^-1^ ammonia) was exfoliated in each run. After exfoliation, the obtained suspension was left to settle by gravity overnight. 3 mL of the supernatant was then taken and centrifuged for 30 min at 5500 rpm for a mild size selection^5^. See Supplementary Figs. S1 for an exemplary visualization of the suspensions from the various stages of the LPE process.

The resulting supernatant was then further characterized using UV–Vis spectroscopy (Jasco UV-670 UV–VIS Spectrophotometer, polymer cuvettes, >230 nm), always incl. empty solvent background removal from the UV–Vis spectrum. The concentrations were determined from UV–Vis absorbance spectra via the absorption at a wavelength of 660 nm. A value of 2460 L·g^-1^·m^-1^ was chosen for the absorption coefficient^14^. This value is considered relatively low and represents a modest estimate of our results to compare them with the results from other studies. We note that for the relative comparison of concentrations within our study, the chosen absorption coefficient value does not change the relative relation of values to each other. To benchmark our result to the state-of-the-art, we also performed benchmark exfoliations in NMP and DMF (Fig. 1a). When graphene concentrations were too high for UV–Vis measurements, samples were diluted in water and measured immediately after dilution with water.

An exemplary measurement of pH for the IPA-water co-solvent system with various IPA-water concentrations and various NH_3_ concentration is shown in Supplementary Fig. 7.

For TEM, three drops of the supernatant were drop-cast onto lacey carbon TEM grids and left to dry naturally. For selected samples, AFM samples were prepared by dropping about 50 µL of the supernatant onto a heated (60 °C) 90 nm SiO_2_-coated silicon wafer. For XPS, selected suspensions were dried in an oven at 60 °C on a polytetrafluoroethylene (PTFE) film, and the resulting powder was used for XPS analysis. For further details on TEM, AFM, XPS, and Zeta potential measurements see Supplementary Information.

## Supplementary information

Supporting Information

## Data Availability

All relevant data are available from the authors.
