# Supplementary material for: Liquid phase exfoliation of graphene using ammonia as an easy-to-remove additive in low-boiling organic-water co-solvent suspensions
Source: Commun Chem. 2025 May 23;8:161. doi: 10.1038/s42004-025-01517-y (PMC12102308; doi:10.1038/s42004-025-01517-y)
Supplement: Supplementary file 1 — Supporting Information [file 42004_2025_1517_MOESM1_ESM.pdf]

Supplementary Information to:

Liquid phase exfoliation of graphene using ammonia as an easy-to-remove additive in low-boiling organic-water co-solvent suspensions

Martin Nastran,[1] Paul Peschek,[1] Izabela Walendzik,[1,2] Jakob Rath,[1] Bernhard Fickl,[1] Jasmin S. Schubert,[1] Wolfgang Ipsmiller,[3] Andreas Bartl,[3] Gerd Mauschitz,[3] Gabriel Szabo,[4] Richard A. Wilhelm,[4] Jochen Schmidt,[5] Dominik Eder,[1,*] Bernhard C. Bayer[1,*]

[1]Technische Universität Wien (TU Wien), Institute of Materials Chemistry, Getreidemarkt 9/165, A-1060 Vienna, Austria

[2]Wrocław University of Science and Technology, Department of Process Engineering and Technology of Polymer and Carbon Materials, Gdańska 7/9, 50-344 Wrocław, Poland

[3]Technische Universität Wien (TU Wien), Institute of Chemical, Environmental and Bioscience Engineering, Getreidemarkt 9/166-1, A-1060 Vienna, Austria

[4]Technische Universität Wien (TU Wien), Institute of Applied Physics, Wiedner Hauptstrasse 8-10/134, A-1040 Vienna, Austria

[5]carbon-solutions Hintsteiner GmbH, Kirchengasse 1, A-8644 Mürzhofen, Austria

*Corresponding authors: bernhard.bayer-skoff@tuwien.ac.at (Bernhard C. Bayer) dominik.eder@tuwien.ac.at (Dominik Eder)

Supplementary Table 1. Information on azeotropes and boiling points of the studied co-solvent mixtures.[1,2] Importantly, none of the studied, low boiling mixtures exhibits an increase of the boiling point compared to water (boiling point: 100 °C), as no mixture exhibits a negative azeotrope. Also, all studied mixtures have boiling points lower than the typical benchmark solvents DMF (153 °C) and NMP (203 °C).

**Mixture:** Water +	**Boiling point (°C)**	**Behaviour**
tert-Butanol	83	positive azeotrope
Ethanol	78	positive azeotrope
Methanol	65	no azeotrope
Isopropanol	83	positive azeotrope
Propanol	97	positive azeotrope
Acetone	56	no azeotrope
Tetrahydrofuran	66	positive azeotrope
Acetonitrile	82	positive azeotrope

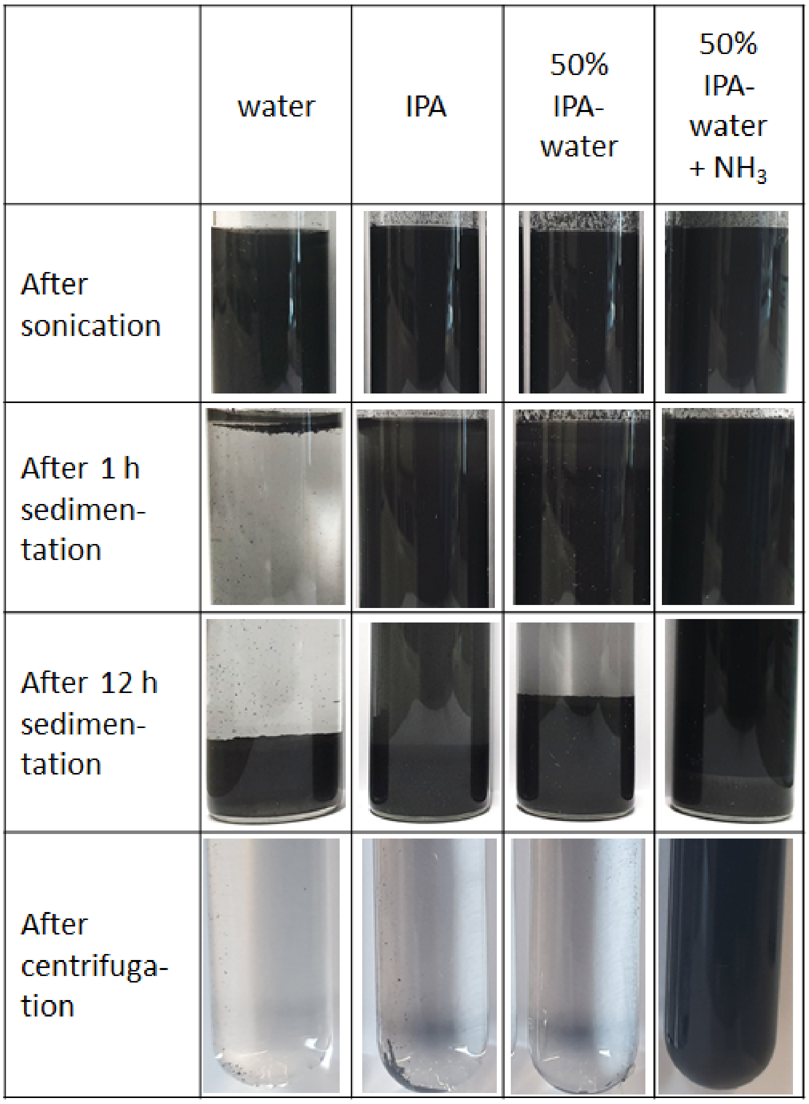

Supplementary Figure 1. Photographs of obtained suspensions in neat water, neat IPA, neat co-solvent mixture 50% IPA-water without NH_3 and optimized co-solvent mixture 50% IPA-water with 50 mmol·L^{-1} NH_3 directly after sonication, after different sedimentation times and after centrifugation.

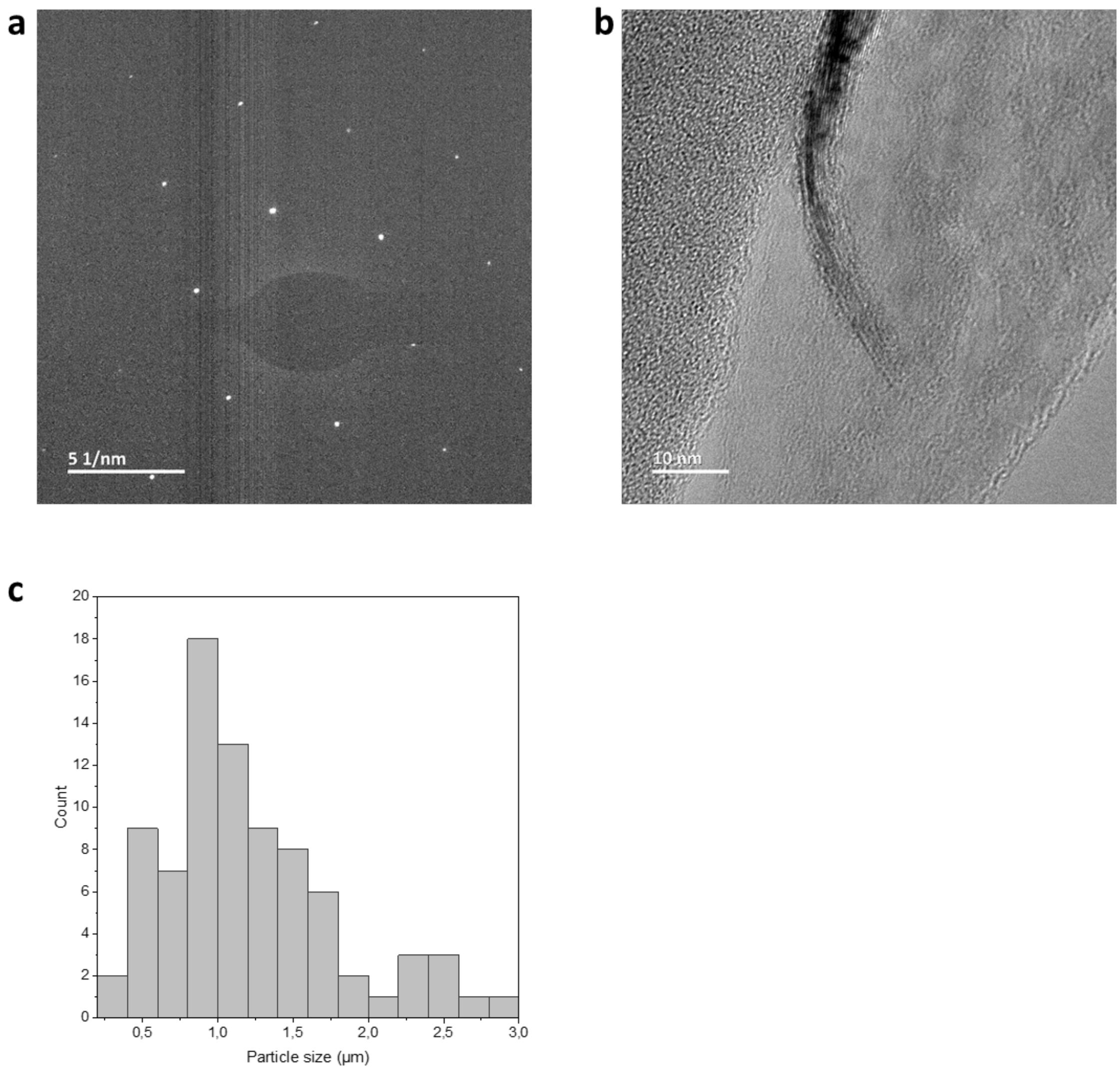

Supplementary Figure 2. TEM images of LPE few-layer graphene flakes from IPA-water 50% with 50 mmol·L^{-1} NH_3 LPE after centrifugation. For overview bright-field TEM image see Figure 3b. (a) depicts a selected area diffraction (SAED) pattern of a graphene flake. Notably, the sharp six-fold SAED pattern confirms a high-quality graphene lattice of the LPE graphene nanoflake. (b) shows a bright-field TEM image of a cross-sectional view of a graphene nanosheet, which allows us to measure nanosheet thickness and count layer number in this few-layer nanosheet. (c) shows a histogram of lateral flake size distribution measured from TEM images such as in Figure 3b using longest axis of flakes. TEM was measured in a FEI Tecnai F20 at 200 kV electron acceleration voltage, acquiring bright-field TEM and SAED data.

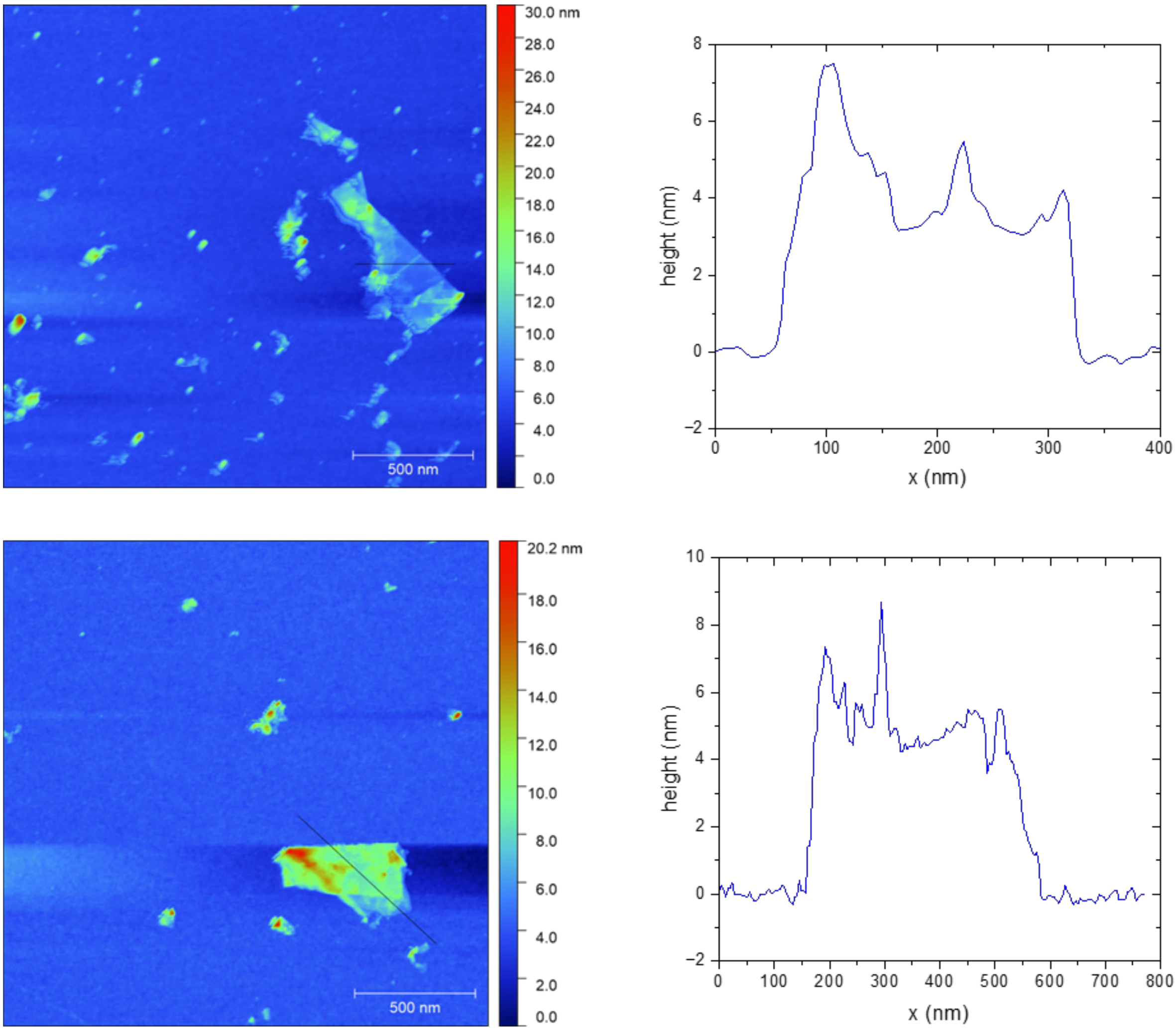

Supplementary Figure 3. AFM images (left) of LPE few-layer graphene flakes from IPA-water 50% with 50 mmol·L^{-1} NH_3 LPE after centrifugation. Corresponding height profiles (right) drawn along the marked lines in the AFM images. AFM was measured in tapping mode using an Asylum Research Cypher AFM with Olympus OMCL-AC240TSR3 tips.

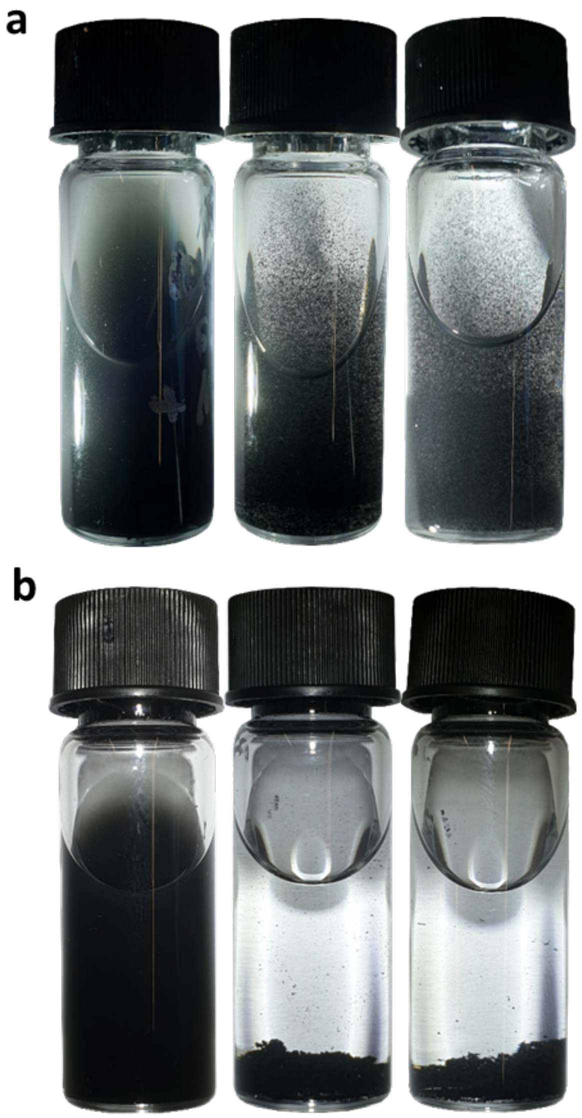

Supplementary Figure 4. Suspensions of LPE graphene from IPA-water 50% with 50 mmol·L^{-1} NH_3 LPE (a) immediately after production (left column) and immediately after control experiments by neutralizing pH using acetic acid (middle column) or hydrochloric acid (right column). (b) Same vial as in (a) after sedimentation over night.

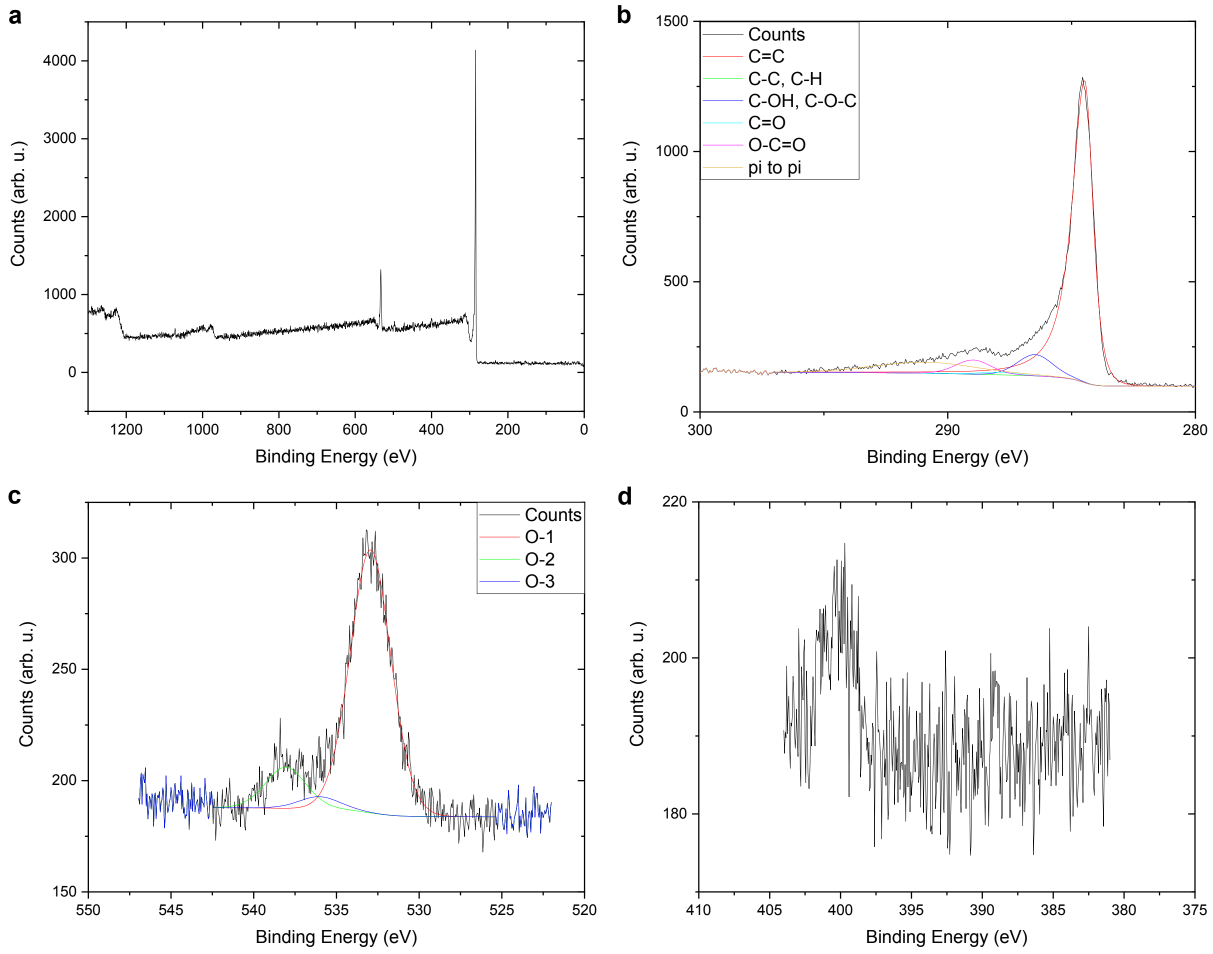

Supplementary Figure 5. (a) XPS survey spectrum of the LPE graphene (IPA-water 50% with 50 mmol·L^{-1} NH_3 after centrifugation) after drying, (b) C1s spectrum, (c) O1s spectrum and (d) N1s spectrum.

XPS

The chemical states of the present elements are evaluated by XPS (Supplementary Figure 5). The survey (Supplementary Figure 5a) shows the expected species: C1s (284 eV) and O1s (530 eV) from the adventitious carbon. The C1s detail spectra (Supplementary Figure 5b), confirms the formation of graphitic carbon as main component (284 eV), showing the typical signal asymmetry.[3,4] The other four weak peaks in C1s centered between 284 to 290 eV correspond to sp^3 hybridized carbon, which we ascribe to graphene nanosheet edges decorated

with adsorbed oxygen, as well as adventitious carbon contamination from sample storage and transport in ambient. Correspondingly, the O1s spectrum (Supplementary Figure 5c) can be deconvoluted into three subpeaks, which could be attributed to the presence of different oxygen functionalities such as C-O, C=O and O=C-O, respectively.[5,6] Such small amounts of oxygen (~6 atom-%) are typical for graphene produced via the LPE route and assumed to be due to flake edge decoration and adventitious carbon adsorption.[7–9] Additionally, even though in the survey no nitrogen was detectable we performed a detail spectra of the N1s region (Supplementary Figure 5d), showing – after many scans – only a negligible amount of N detected. This places N content near XPS detection limit <<1 atom-%.

All XPS measurements were carried out on a custom-built SPECS XPS-spectrometer equipped with a monochromatised Al-K_α X-ray source (μFocus 350) and a hemispherical WAL-150 analyser (acceptance angle: 60°). All samples were mounted onto the sample holder using double-sided carbon tape. Pass energies of 100 eV and 30 eV and energy resolutions of 1 eV and 100 meV were used for survey and detail spectra respectively (excitation energy: 1486.6 eV, beam energy and spot size: 70 W onto 400 μm, angle: 51° to sample surface normal, base pressure: $5x10^{-10}$ mbar, pressure during measurements: $2x10^{-9}$ mbar). Data analysis was performed using CASAxps software, employing transmission corrections (as per the instrument vendor's specifications), Shirley backgrounds[10] and Scofield sensitivity factors.[11] All content values shown are in units of relative atomic percent (atom-%), where the detection limit in survey measurements usually lies around 0.1-1 atom-%, depending on the element. Assignment of different components was primarily done using references.[12,13]

Zeta potential

Zeta potential measurements were conducted for the chosen samples on a Litesizer™ DLS 500 from Anton Paar using 3 mL quartz-glass cuvettes. Prior to measurement, a clean and dry cuvette was filled with sample and let sit for 5 min for conditioning, before discarding this fill. 800 µL of fresh sample was added and a Univette electrode measurement head (Mat. No. 183578) and spacer was carefully attached to the cuvette, checking for the absence of air bubbles due to filling, before fully immersing both electrodes. The key measurement and substance parameters (including refractive index, viscosity, and relative permittivity for the solvent) for the mixture were derived via linear interpolation between known values from a device substance database. After granting a temperature equilibration time of 30 s each, measurements were conducted at 25 °C, whereat low numbers of just around 100 process runs per sample were sufficient to reach autorun quality criteria, confirming good measurement properties. Gathered data was processed using a custom approximation and a Henry factor of 1.3, individually calculated within the device's Kalliope software for our sample system.

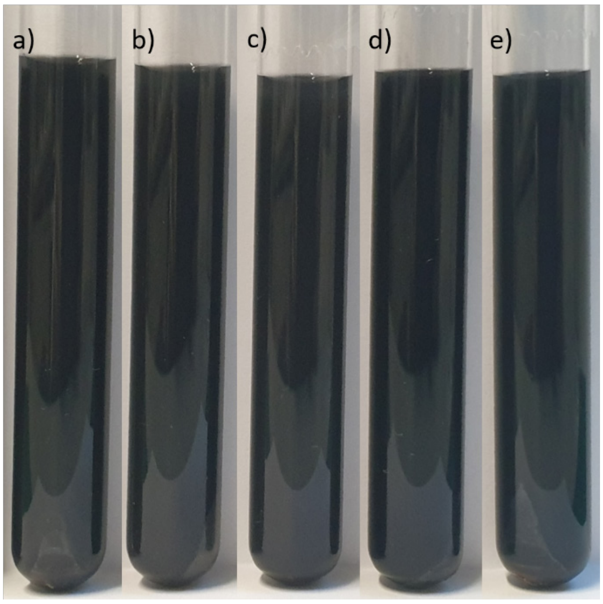

Supplementary Figure 6. Photographs of exfoliated samples from 50 % IPA-water with 50 mmol·L^{-1} NH_3 after centrifugation comparing 5 different graphite sources: (a) pristine graphite flakes (325 mesh, <45 µm) from Alfa Aesar used in all other experiments in this study, (b) Natural graphite UF4 supplied by Graphit Kropfmühl GmbH, (c) TIMCAL TIMREX® KS6 Synthetic Graphite, (d) TIMCAL TIMREX® KS75 Primary Synthetic Graphite, (e) TIMREX® SFG 75 Graphite. We find that our optimized recipe enables stable exfoliation of all tested graphite sources, underscoring the generality of our findings beyond any particular graphite source.

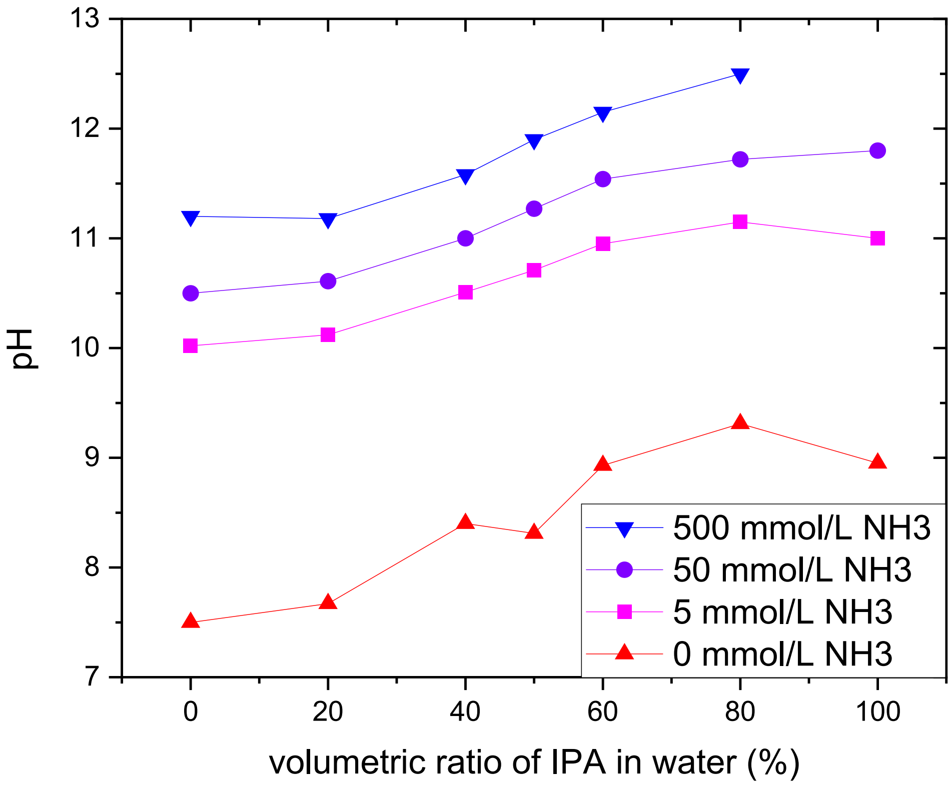

Supplementary Figure 7. pH of IPA-water mixtures (0 to 100%) with 0, 5, 50 and 500 mmol·L^{-1} NH_3. pH was measures using a Mettler Toldedo pH electrode setup. Calibration was performed using puffers of pH 4, 7 and 10 and pH interpolated using internal linear regression.

Supplementary References

1. Lide, D. R. *CRC Handbook of Chemistry and Physics 88TH Edition 2007-2008*. (2007).
2. Fan, Z. D., Zhang, X. B., Zhao, L. Y., Cai, W. F. & Wang, F. M. Study on the Separation of Azeotrope of Tetrahydrofuran-Water Using a Combined Method of Extractive and General Distillation. *Adv. Mater. Res.* **803**, 149–152 (2013).
3. Mezzi, A. & Kaciulis, S. Surface investigation of carbon films: from diamond to graphite. *Surf. Interface Anal.* **42**, 1082–1084 (2010).
4. Morgan, D. J. Comments on the XPS Analysis of Carbon Materials. *C* **7**, 51 (2021).
5. Miller, D. J., Biesinger, M. C. & McIntyre, N. S. Interactions of CO2 and CO at fractional atmosphere pressures with iron and iron oxide surfaces: one possible mechanism for surface contamination? *Surf. Interface Anal.* **33**, 299–305 (2002).
6. Barr, T. L. & Seal, S. Nature of the use of adventitious carbon as a binding energy standard. *J. Vac. Sci. Technol. Vac. Surf. Films* **13**, 1239–1246 (1995).
7. Güler, Ö., Tekeli, M., Taşkın, M., Güler, S. H. & Yahia, I. S. The production of graphene by direct liquid phase exfoliation of graphite at moderate sonication power by using low boiling liquid media: The effect of liquid media on yield and optimization. *Ceram. Int.* **47**, 521–533 (2021).
8. Liu, M. *et al.* One-step chemical exfoliation of graphite to ~100% few-layer graphene with high quality and large size at ambient temperature. *Chem. Eng. J.* **355**, 181–185 (2019).
9. Janowska, I. *et al.* Microwave synthesis of large few-layer graphene sheets in aqueous solution of ammonia. *Nano Res.* **3**, 126–137 (2010).
10. Shirley, D. A. High-Resolution X-Ray Photoemission Spectrum of the Valence Bands of Gold. *Phys. Rev. B* **5**, 4709–4714 (1972).

11. Scofield, J. H. Hartree-Slater subshell photoionization cross-sections at 1254 and 1487 eV. *J. Electron Spectrosc. Relat. Phenom.* **8**, 129–137 (1976).

12. Naumkin, A. *et al.* NIST X-ray Photoelectron Spectroscopy Database, NIST Standard Reference Database 20, Version 3.4. *National Institute of Standards and Technology: Gaithersburg, MD* **20899**, (2003).

13. Beamson, G. & Briggs, D. High Resolution XPS of Organic Polymers. *Sci. ESCA300 Database* (1992).
